# The Clinical Efficiency and the Mechanism of Sanzi Yangqin Decoction for Chronic Obstructive Pulmonary Disease

**DOI:** 10.1155/2021/5565562

**Published:** 2021-06-10

**Authors:** Mengqi Wang, Wenwen Gu, Fuguang Kui, Fan Gao, Yuji Niu, Wenwen Li, Yaru Zhang, Lijuan Guo, Shengnan Geng, Gangjun Du

**Affiliations:** ^1^School of Pharmacy and Chemical Engineering, Zhengzhou University of Industry Technology, Xinzheng, Henan Province 451150, China; ^2^Institute of Pharmacy, Pharmaceutical College of Henan University, Jinming District, Kaifeng, Henan Province 475004, China

## Abstract

This work is carried out to evaluate the clinical efficacy of Sanzi Yangqin decoction (SZYQD) treating chronic obstructive pulmonary disease (COPD) and to analyze its mechanism. The clinical efficacy of SZYQD treating COPD was evaluated by meta-analysis, and its mechanism was analyzed by network pharmacology. Molecular docking validation of the main active compounds and the core targets was performed by AutoDock vina software. A cigarette smoke (CS) and LPS-induced COPD model in ICR mice was constructed to confirm the effects of luteolin on COPD. Results showed that SZYQD has a greater benefit on the total effect (OR = 3.85, 95% CI [3.07, 4.83], *P*=1) in the trial group compared with the control group. The percentage of forced expiratory volume in one second (FEV1%) (MD = 0.5, 95% CI [0.41, 0.59], *P* < 0.00001) and first seconds breathing volume percentage of forced vital capacity (FEV1%/FVC) were improved (MD = 5.97, 95% CI [3.23, 8.71], *P* < 0.00001). There are 27 compounds in SZYQD targeting 104 disease targets related to COPD. PPI network analysis indicated that EGFR, MMP9, PTGS2, MMP2, APP, and ERBB2 may be the core targets for the treatment of COPD. Molecular docking demonstrated that luteolin in SZYQD showed the strongest binding activity to core targets. Experimental results revealed that the expression of COPD-related targets in lung tissue was significantly increased in the COPD group and was improved in the luteolin group. Our data indicated that SZYQD has a curative effect on COPD and luteolin is a candidate compound for COPD treatment by regulating EGFR, MMP9, PTGS2, MMP2, APP, and ERBB2.

## 1. Introduction

Chronic obstructive pulmonary disease (COPD), caused mainly by cigarette smoking, is characterized by chronic airway inflammation and persistent airflow limitation and is the fourth leading cause of death in the world [1]. A recent study identified that there are about 100 million patients with COPD in China, which not only have a heavy social and economic burden but also seriously affect their quality of life [2]. The treatment of COPD has made enormous progress in the past. Inhalation of long-acting bronchodilators or glucocorticoids is the primary treatment for COPD, which has been confirmed to have beneficial effects in reducing exacerbation rates and boosting health conditions [3–5]. Despite vast improvements in the treatment of COPD, patients still suffer from cough with sputum production, dyspnea, wheezing, and chest tightness. Therefore, seeking other effective treatments has always remained a continuing problem for COPD.

**Figure 1:**
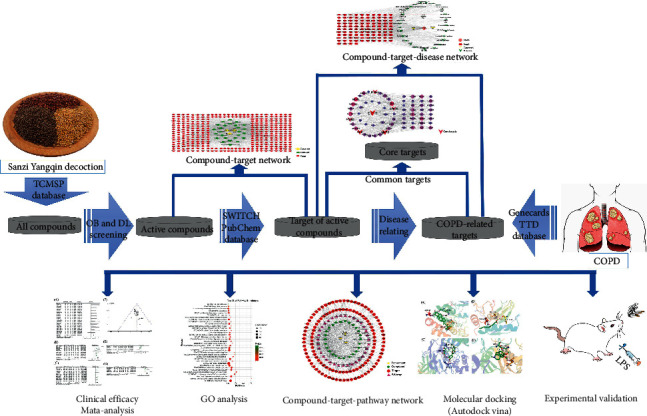
The flow chart of this whole analysis for this study.

Traditional Chinese Medicine (TCM) has defended Chinese people's health for thousands of years ago. TCM plays a significant role in remodeling the airway and reducing airway hyperresponsiveness in COPD [6]. Among them, Sanzi Yangqin decoction (SZYQD) is a commonly used multiherb TCM with a satisfactory efficacy, such as antitussive, expectorant, and antiasthmatic [7]. This formula was first described in Han's Medicine Theory, which is composed of white mustard, radish, and perilla seed [8–10]. White mustard seed is the dry mature seed of *Sinapis alba* L. of the cruciferous plant. It has the pharmacological effects of antitussive, expectorant, antiasthmatic, anti-inflammatory, and analgesic effects [11, 12]. It is commonly used in the treatment of pneumonia and bronchial asthma [13]. Radish seed is the dry mature seed of *Raphanus sativus* L., which contains alkaloids, glucosinolates, isothiocyanates, flavonoids, volatile oil, fatty oil, protein, polysaccharides, etc. [14]. It has the effect of eliminating bloating, reducing Qi, and resolving phlegm [15]. Perilla seed is the dry ripe fruit of *Perilla frutescens* L. Britt. Its leaves, stems, and fruits can be used as medicine. It has the function of reducing gas and eliminating phlegm, relieving asthma, and moistening the intestines [16]. However, its efficacy has not been comprehensively and systematically evaluated. Therefore, this paper will use meta-analysis to evaluate the efficacy of SZYQD on COPD through the study of a large number of clinical data. Currently, network pharmacology analysis has been widely used to evaluate the interactions between proteins and molecules in biological systems to study the mechanisms of TCM [17, 18], which provides a new and powerful method for these multitarget drugs. The combination of classical pharmacology and systems pharmacology analysis may provide a better strategy for identifying new therapeutic uses and mechanisms for TCM. Therefore, this study will also perform network pharmacology and animal experiments to elucidate the mechanism of SZYQD on COPD. A workflow of the study is summarized as shown in Figure 1.

## 2. Materials and Methods

### 2.1. Literature Search

We systematically searched the Cochrane Library, PubMed, Web of Science, Embase, and CNKI from 2005 to 2020 for eligible articles and included them in the meta-analysis. The keywords that were used are as follows: Sanzi Yangqin tang, Sanzi Yangqin decoction, SZYQD, chronic obstructive pulmonary disease, and COPD. Besides, we also performed other searches and checked reference lists of relevant reviews and eligible randomized controlled trials to ensure a comprehensive search.

### 2.2. Inclusion and Exclusion Criteria

The inclusion criteria were as follows: (1) randomized, clinical trial; (2) control group for routine western medicine treatment and experimental group for SZYQD based on other medicines or SZYQD alone; (3) basis of disease diagnosis being nomenclature and diagnostic criteria for COPD; (4) clinical efficacy, lung function index of FEV1% and FEV1%/FVC, and blood gas analysis index of the PaO_2_ and PaCO_2_ as outcome indicators; and (5) conforming to the ethical and moral treatment standard. Meanwhile, exclusion criteria include the following: (1) nonrandomized trials; (2) experiments on animals; (3) review; (4) no clear diagnostic criteria or not meeting inclusion criteria; (5) lack of required data for meta-analysis; (6) the sample size being less than 40; and (7) no complete evaluation of efficacy.

### 2.3. Data Extraction

Two independent reviewers searched the literature according to the titles and abstracts and the established strategy, then extracted data from the included studies, performed duplicate checking, and compared the results carefully. Disagreements were resolved by a third investigator to avoid bias. The name of the first author and year of publication were extracted for identification in our analysis.

### 2.4. Quality Assessment

We assessed the quality of the literature according to the Cochrane Collaboration's Bias risk assessment tool. The main assessment areas are as follows: random sequence generation, allocation concealment, the blinding method for patients/researchers and outcomes assessors, incomplete outcome data, selective reporting, and other sources of bias. The results were judged as “low risk”, “high risk”, and “unclear”.

### 2.5. Screening of Active Compounds and Targets

The main chemical compounds of white mustard, radish, and perilla seed in SZYQD were retrieved from TCM Systems Pharmacology Database (TCMSP, https://tcmspw. com/tcmspsearch.php) [19], which were mainly filtered by integrating oral bioavailability (OB) > 30% and drug-likeness (DL) > 0.15. The targets of the active compounds in SZYQD were obtained from the TCMSP database, PubChem database (https://pubchem.ncbi.nlm.nih.gov/), and STITCH (http://stitch.embl.de/) [20]. GeneCards Database (https://www.genecards.org/) and Therapeutic Target Database (TTD, https://db.idrblab.org/ttd/) were used to collect the therapeutic targets related to COPD. COPD-related targets and drug targets were mapped in Venny 2.1.0 to select the common targets. In order to explicit the interaction between the potential disease targets of SZYQD, the PPI networks were constructed from STRING [21] (https://string-db.org/). The limited species was “*Homo sapiens*”, and the minimum confidence score was ≥0.700. The core targets were screen with the network analyzer plugin in Cytoscape 3.7.2.

### 2.6. Gene Ontology and KEGG Pathway Enrichment

In order to elucidate the action mechanisms of SZYQD on COPD, DAVID 6.8 (https://david.ncifcrf.gov/) [22] was used to analyze GO biological processes and KEGG pathways. GO biological processes and KEGG pathways with *P* value < 0.05 and Count >7 were employed. Gene set enrichment analyses were performed by using the OmicShare tools (http://www.omicshare.com/tools/), which visualized the enrichment analysis results.

### 2.7. Network Construction and Analysis

To facilitate the visualization of multiple-target effects of SZYQD and COPD interrelation, compound-target, disease-target-compound, and compound-target-pathway networks were constructed by Cytoscape 3.7.2. In these networks, we used nodes to stand for the components, compounds, targets, and diseases, and the edges between the two nodes represented their interaction.

### 2.8. Molecular Docking Verification

Molecular docking was carried out between the top 6-degree value of active compounds in the disease-compound-target network and the top 6-degree value of the target genes in the PPI network. The mol2 format structure files of chemical compounds were obtained from the TCMSP database, and the crystal structures of core targets from the RCSB Protein Data Bank (PDB, http://www.pdb.org/) were collected. The processed crystal structures of core targets and structures of active compounds were imported into AutoDock vina software [23] for molecular docking.

### 2.9. Materials

Antibodies against *α*-SMA (1 : 1000), TGF-*β*1 (1 : 1000), EGBB2 (1 : 1000), MMP2 (1 : 1000), MMP9 (1 : 1000), PTGS2 (1 : 1000), APP (1 : 500), EGFR (1 : 1000), and *β-*actin (1 : 1000), as well as HRP goat anti-mouse antibody (1 : 10000) and FITC goat anti-mouse antibody (1 : 1000), were obtained from BD Pharmingen. Sanhua cigarette was provided by Hunan Tobacco (Changsha, China). LPS (lipopolysaccharide) was purchased from Sigma Chemical Co. (St. Louis, MO, United States). Reactive Oxygen Species Assay Kit (ROS, DCFH-DA) and DAPI were obtained from Beijing Solarbio Technology Co., Ltd. (Beijing, China). Mice quantitative ELISA kits, IL-6 (M6000 B) and TGF-*β*(MB100 B), were obtained from R&D Systems (Minnesota, USA).

Luteolin (purity > 98% via HPLC, batch number: L107328) was purchased from Lianshuo Biotechnology Co., Ltd. (Shanghai, China). Dexamethasone Tablets (75 mg/tablet, batch number: 191067) were purchased from Zhangzhongjing Pharmacy (Zhengzhou, China). Ix53 inverted fluorescence microscope and Cx31 upright microscope were purchased from OLYMPUS company, Japan. G: BOX multifunctional gel imaging system was purchased from Syngene, UK. The animal respiratory metabolic measurement system was obtained from Sable Systems International, United States.

### 2.10. Animals

Seven-week-old female ICR mice were obtained from Henan Provincial Medical Laboratory Animal Center. All mice were housed in individually ventilated cages (lights on 7: 00 AM to 7: 00 PM). Animals were fed standard rodent chow and water. All animal procedures were approved by the Animal Experimentation Ethics Committee of Henan University (permission number HUSAM 2016-288), and all procedures were performed in strict accordance with the Guide for the Care and Use of Laboratory Animals and the Regulation of Animal Protection Committee to minimize suffering and injury. Animals were euthanized via carbon dioxide overdose based on experimental need. Standard rodent chow was purchased from Henan Provincial Medical Laboratory Animal Center (Zhengzhou, China), under License No. SCXK (YU) 2015–0005, Certificate No. 41000100002406.

### 2.11. CS and LPS-Induced COPD Model

The animals were divided into six groups (*n* = 10/group): group one, control group; group two, CS + LPS group; group three, CS + LPS/dexamethasone group (positive control; 1 mg/kg body weight); and groups four to six, CS + LPS/luteolin group (5, 10, 20 mg/kg body weight). The COPD mice model was established where mice received an intraperitoneal injection of LPS (750 ng/kg solved in 50 *μ*L saline) once weekly for 6 weeks. The next day after LPS injection, the mice were exposed to cigarette smoke (2 cigarettes each hour, 1 hour each time, twice a day, and 6 days per week) in a whole-body exposure system (30 cm × 40 cm × 60 cm's chamber) for 6 weeks. Following the first LPS injection, treated mice received therapeutic drugs via intragastric administration once daily for 6 weeks. Food and water were provided ad libitum during the study. The health of the mice was monitored daily, and body weights were measured weekly. Lung function was analyzed weekly by tidal volume using the animal respiratory metabolic measurement system. One week after the last CS exposure, mice were sacrificed under anesthesia with pentobarbital sodium (90 mg/kg), and a part of each lung was preserved in 10% buffered formalin and routinely embedded in paraffin. Lung sections were stained with hematoxylin and eosin (H&E), and the pathological score was determined as previously reported [24].

### 2.12. Broncho Alveolar Lavage Fluid (BALF) Collection and Analysis

The BALF collection was carried out as described [25]. Briefly, 1 mL 1 × PBS was injected and withdrawn intratracheally three times. The centrifuged inflammatory cells (white cells, neutrophils, and lymphocytes) in collected BALF were resuspended and counted via a hemocytometer. The supernatants were kept at −80°C before the evaluation of IL-6, TGF-*β* cytokines, and ROS using ELISA kits following the manufacturer's specification.

### 2.13. Immunofluorescence Staining

Lung tissue sections were treated with blocked with 5% BSA for 30 min at room temperature and incubated with anti-EGBB2, anti-MMP2, anti-MMP9, anti-PTGS2, anti-APP, anti-EGFR, and secondary antibody (FITC goat anti-mouse IgG). Then, the sections were fixed with antifluorescence quencher, observed, and photographed under the fluorescence microscope. Fluorescence intensity was quantified using ImageJ software (NIH, Bethesda, MD, USA).

### 2.14. Western Blot Analysis

Proteins were extracted and separated via 12% sodium dodecyl sulfate-polyacrylamide gel electrophoresis, electroblotted onto nitrocellulose membranes, and probed with antibodies against *α*-SMA, TGF-*β*1, EGBB2, MMP2, MMP9, PTGS2, APP, EGFR, and *β*-actin. Band density was quantified using ImageJ software and normalized to the corresponding control group.

### 2.15. Statistical Analyses

Statistical analyses in the meta-analysis were performed using RevMan 5.3. The SMD and corresponding 95% CI were calculated to evaluate the effect of SZYQD on COPD. The heterogeneity was calculated using *I*^2^ statistics. If high heterogeneity (*I*^2^ > 50%) was observed, random-effect models were applied; otherwise, fixed-effect models were used. Funnel plots were used to identify potential publication bias. The data were statistically analyzed using GraphPad Prism, Version 5.0 (San Diego, CA, USA), and presented as the mean ± SD. The differences between the two groups were evaluated using a *t*-test. A *P* value of less than 0.05 was considered statistically significant.

## 3. Results

### 3.1. Search Results and Quality Assessment

**Figure 2:**
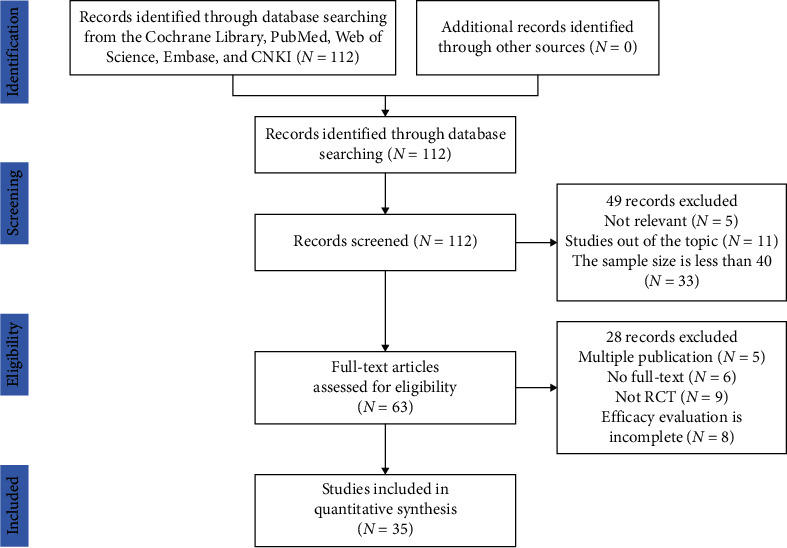
Flow diagram of the study selection process. Search strategy and flow chart of the screened, excluded, and analyzed articles.

**Figure 3:**
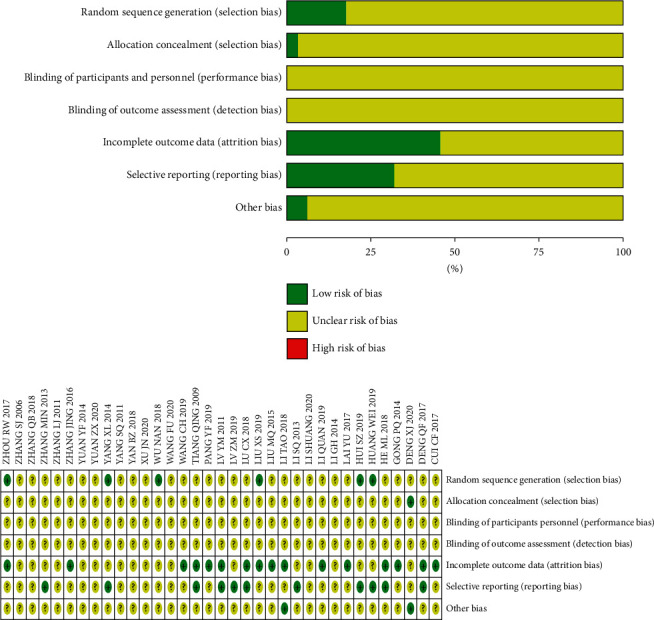
Quality assessment of 35 available studies. (a) Risk of bias graph: review authors' judgments about each risk of bias item presented as percentages across all included studies. (b) Risk of bias summary: review authors' judgments about each risk of bias item for each included study.

The search process is shown in Figure 2. All databases generated 112 potentially relevant studies in the initial search. Among them, 77 studies were excluded due to irrelevant content or not up to inclusion criteria after reading the titles and abstracts. 35 studies were subsequently selected for the final analysis. A total of 3730 patients from 35 included studies containing 1847 control groups and 1883 treatment groups were included in this meta-analysis. The detailed data extracted from these included articles are listed in Table 1. Quality assessment of 35 available studies was performed by using the Risk Assessment Tool of the Cochrane Library. The risk of inclusion literature is shown in Figure 3.

### 3.2. Clinical Efficacy, Lung Function, and Blood Gas Analysis

**Figure 4:**
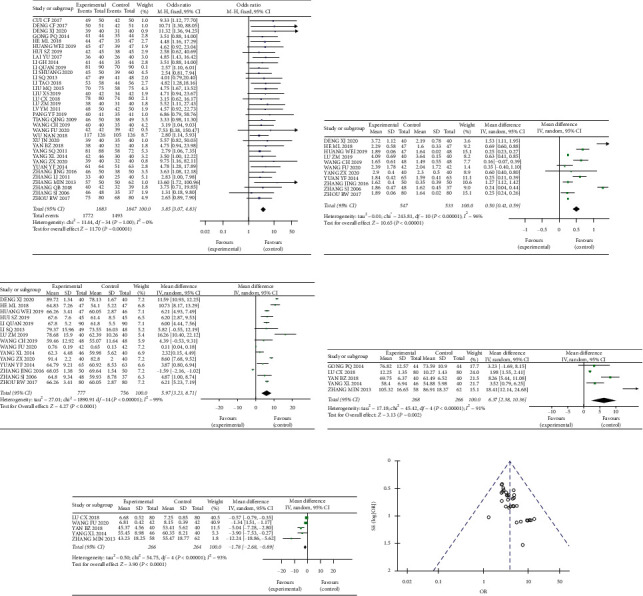
Sanzi Yangqin decoction has a curative effect on COPD. (a) Comparison of total effect between the experimental group and the control group for COPD. (b, c) Forest plot analyzing lung function of COPD patients with SZYQD: FEV1; FEV1/FVC%. (d, e) Forest plot analyzing blood gas of COPD patients with Sanzi Yangqin decoction: PaO_2_; PaCO_2_. (f) Funnel plot analyzing the bias of the total effect in the treatment of COPD by Sanzi Yangqin decoction.

All 35 studies, including a total of 3730 patients, reported the total effect of SZYQD on COPD. Due to the low heterogeneity (*I*^2^ = 0%), we used a fixed-effect model to analyze the results. Compared with the control group, the clinical symptoms in COPD patients were improved (OR = 3.85, 95% CI [3.07, 4.83], *P*=1, Figure 4(a)) in the trial group. 11 of the 35 trials used FEV1% to evaluate the effect of SZYQD in COPD patients. A random-effect model was used to analyze the results. The effect size of the 11 trials showed that SZYQD could significantly improve the FEV1% (MD = 0.5, 95% CI [0.41, 0.59], *P* < 0.00001, *I*^2^ = 96%, Figure 4(b)). 15 of the 35 trials used the FEV1%/FVC to assess the improvement in lung function. The effect size of the six trials showed that SZYQD could significantly improve the FEV1%/FVC (MD = 5.97, 95% CI [3.23, 8.71], *P* < 0.00001, *I*^2^ = 99%, Figure 4(c)). PaO_2_ and PaCO_2_ were used to assess the improvement of blood gas, and a random-effect model was used to analyze the results (*I*^2^ = 91%, 93%). The meta-analysis showed that compared with the control group, PaO2 was heightened (MD = 6.37, 95% CI [2.38, 10.36], *P* < 0.00001, Figure 4(d)), but PaCO_2_ was decreased (MD = −1.78, 95% CI [−2.68, −0.89], *P* < 0.00001, Figure 4(e)) in the trial group.

### 3.3. Publication Bias

Funnel plots were made to evaluate publication bias. The result indicated that there is no clear bias in the total effect (Figure 4(f)).

### 3.4. Screening of Active Compounds and Targets in Sanzi Yangqin Decoction

**Figure 5:**
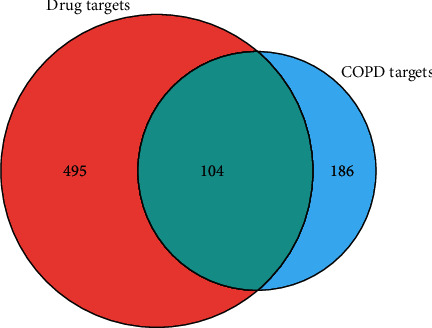
Venn diagram of drug-disease intersection targets. The 290 targets of COPD were mapped to the 599 targets of SZYQD to screen out the 104 common targets.

**Figure 6:**
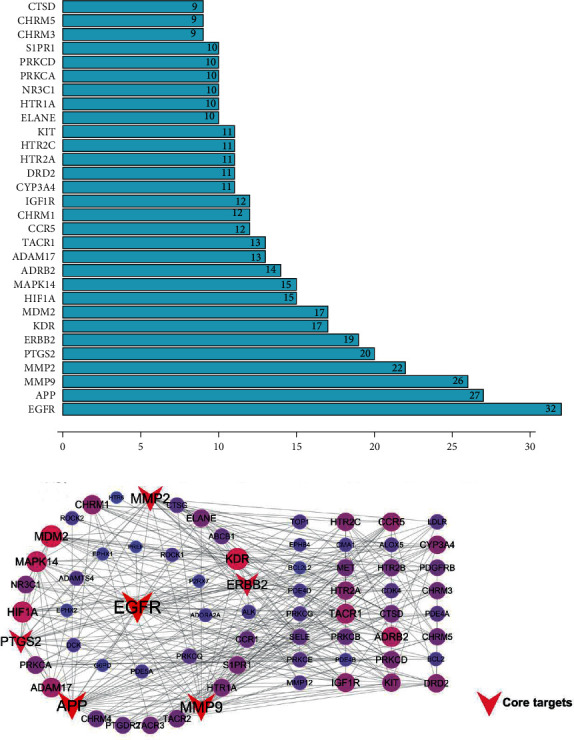
The PPI network constructed by the STRING database and Cytoscape 3.7.2. (a) The number of nodes in the protein interaction network (Top 30). (b) Core targets of Sanzi Yangqin decoction for the treatment of COPD. The top 6 targets as SZYQD's main therapeutic targets, such as EGFR, MMP9, PTGS2, MMP2, APP, and ERBB2, were associated with the development of COPD.

The compounds of white mustard, radish, and perilla seed in SZYQD were retrieved from TcmSPTM. According to the standard of OB ≥ 30%, DL > 0.15. A total of 27 active compounds were obtained for SZYQD, including three compounds in white mustard seed, six compounds in radish seed, and eighteen compounds in perilla seed (shown in Supplementary File 1). We performed target fishing on the 27 active compounds based on databases and collected 1019 drug targets, among which there were 203 in white mustard seed, 290 in radish seed, and 526 in perilla seed (shown in Supplementary File 2). 290 COPD-related targets were found in different databases, 599 drug targets and 290 COPD-related targets were mapped in Venny 2.1.0, and 104 common targets were obtained (Figure 5). PPI network data were imported into Cytoscape 3.7.2, which covered 71 nodes and 316 interaction lines, and the average degree value was 12.9 (Figure 6(a)). 6 core targets, including EGFR, MMP9, PTGS2, MMP2, APP, and ERBB2, were selected according to the descending order of degree value (Figure 6(b)). These targets were associated with the development of COPD and its complications.

### 3.5. GO and KEGG Enrichment Analysis

**Figure 7:**
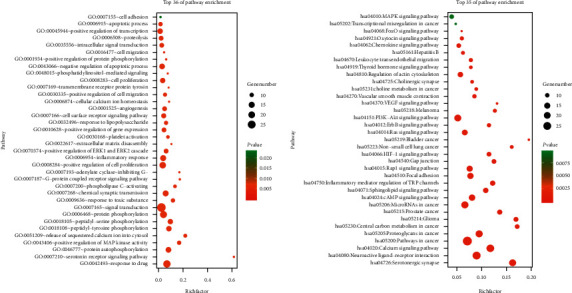
Gene enrichment analyses performed by DAVID and visualized by OmicShare. (a) GO biological process. The results showed that vital targets have a very strong correlation with physiological mechanisms, such as the serotonin receptor signaling pathway, protein autophosphorylation, positive regulation of MAP kinase activity, and release of sequestered calcium ion into the cytosol. (b) KEGG enrichment analysis. The main pathways were the cancer pathway, calcium signaling pathway, PI3K-Akt signaling pathway, and Ras signaling pathway. The vertical axis is the pathname and the horizontal axis is the Rich Factor value. The larger the *P* value, the higher the channel enrichment; the size of the point indicates the number of enriched targets; the color of the dots is red to green and the value is from small to large.

104 predicted targets were mapped to the DAVID database to systematically analyze their GO and KEGG pathway enrichment. 37 GO biological processes and 35 KEGG pathways were obtained. GO biological processes showed that these predicted targets have a very strong correlation with physiological mechanisms, such as serotonin receptor signaling pathway, protein autophosphorylation, positive regulation of MAP kinase activity, and release of sequestered calcium ion into the cytosol. The main pathways were the cancer pathway, calcium signaling pathway, PI3K-Akt signaling pathway, and Ras signaling pathway. Besides, GO biological processes and the KEGG pathway visualized the enrichment analysis results via the OmicShare tools (Figures 7(a) and 7(b)).

### 3.6. Network Analysis

**Figure 8:**
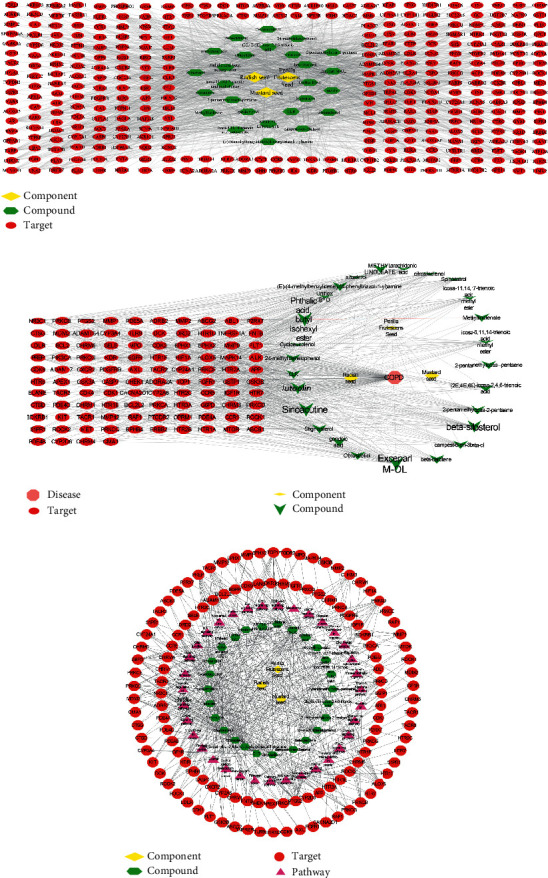
C-T network, D-T-C network, and C-T-P network: (a) a compound-target network, and nodes represent component, compounds, and targets; (b) a disease-target-compound network, red nodes represent the drug-disease intersection targets, green nodes represent the compounds, yellow nodes represent the components, while the pink nodes represent disease, and disease is linked with compounds and targets; (c) a compound-target-pathway network, red nodes represent the drug-disease intersection targets, green nodes represent the compounds, yellow nodes represent the components, while purple nodes represent the pathways. And then, each edge symbolizes the interaction between them.

Compound-target network (C-T network) was constructed via Cytoscape 3.7.2. The nodes represent compounds and targets. The edges represent their interactions (Figure 8(a)). The C-T network embodies 604 nodes and 2650 interaction relationships. To explore the relationships between COPD, their associated targets, and the corresponding compounds, we further established a disease-target-compound network (D-T-C network). As shown in Figure 8(b), the D-T-C network consists of 138 nodes and 632 interaction relationships, and the degree value of active compounds was analyzed (shown in Supplementary File 1). Sinoacutine, exceparl M-OL, phthalic acid, butyl isohexyl ester, luteolin, beta-sitosterol, and stigmasterol may be the main active compounds of SZYQD. To further characterize the molecular mechanism of SZYQD on COPD, a compound-target-pathway network (C-T-P network) was performed based on active compounds, targets, and their corresponding signal pathways. Therefore, 35 signal pathways were selected in the treatment of COPD by SZYQD, constructing the C-T-P network (Figure 8(c)). They interact with 190 nodes and 512 interaction relationships.

### 3.7. Active Compounds and Core Targets Docking Verification

**Figure 9:**
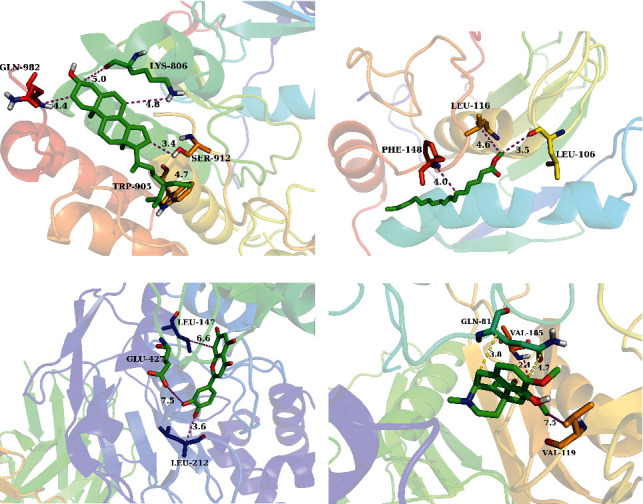
Pattern diagram of molecular docking. (a) Beta-sitosterol and EGFR. (b) Exceparl M-OL and MMP2. (c) Luteolin and MMP9. (d) Sinoacutine and APP.

The crystal structures of EGFR (5y9t), MMP9 (5th9), MMP2 (3ayu), APP (3ktm), ERBB2 (3wlw), and PTGS2 (5kir) were obtained from the RCSB Protein Data Bank (PDB). The crystal structure of each protein was selected based on the best resolution available. The active compounds of SZYQD, such as sinoacutine, exceparl M-OL, phthalic acid, butyl isohexyl ester, luteolin, beta-sitosterol, and stigmasterol, were docked with the targets of EGFR, MMP9, PTGS2, MMP2, APP, and ERBB2. The results showed that sinoacutine, exceparl M-OL, phthalic acid, butyl isohexyl ester, luteolin, beta-sitosterol, and stigmasterol had a good binding ability with EGFR, MMP9, PTGS2, MMP2, APP, and ERBB2 (docking score > 4.25) (Table 2). In particular, luteolin showed the strongest binding activity with EGFR, MMP9, PTGS2, MMP2, APP, and ERBB2 (docking score > 7). Next, a pharmacology experiment was designed to validate the effect of luteolin on COPD induced by CS and LPS. PyMol software was used to display the interaction of the ligand with the receptor-binding site. Beta-sitosterol and EGFR, exceparl M-OL and MMP2, luteolin and MMP9, and sinoacutine and APP were selected for visualization with PyMol software (Figure 9).

### 3.8. Effects of Luteolin on Lung Function and Alveolar Architecture in Mice with COPD

**Figure 10:**
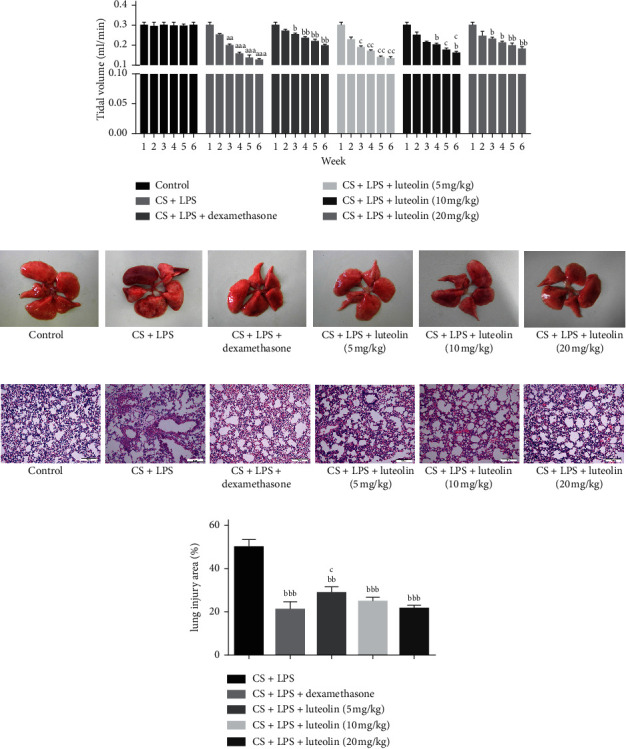
Effects of luteolin on lung function and alveolar architecture in mice with COPD. (a) Tidal volume (TV) in lung function. (b) The whole lung in the naked eye. (c) HE staining of lung tissues. (d) The area of involved lesions in HE staining. The data presents mean ± SD (*n* = 7), the experiments were repeated 3 times, and statistical significance was determined by a *t*-test. ^aa^*P* < 0.01, ^aaa^*P* < 0.001 vs control; ^b^*P* < 0.05, ^bb^*P* < 0.01, ^bbb^*P* < 0.001 vs CS + LPS group; ^c^*P* < 0.05, ^cc^*P* < 0.01 vs dexamethasone.

The tidal volume (TV) was used for detecting lung function. As shown in Figure 10(a), tidal volume was significantly decreased in the CS and LPS group compared with the control group but increased in dexamethasone and luteolin group (tidal volume in the lung function decreased more than 20% among mice). The CS and LPS group exhibited enlarged alveolar space and thinner alveolar septum and destroyed alveolar wall compared with the control group. However, luteolin treatment dose-dependently alleviated CS and LPS-induced lung pathologic changes compared with CS and LPS group (Figures 10(b)–10(d)).

### 3.9. Effects of Luteolin on Inflammatory Cells, Cytokine Levels, and ROS in BALF of Mice with COPD

**Figure 11:**
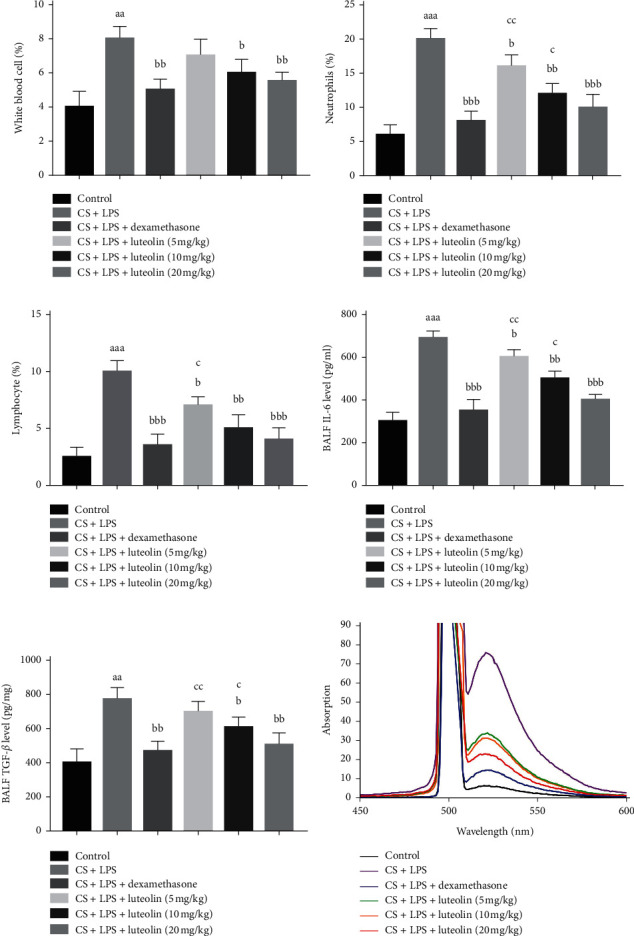
Effects of luteolin on inflammatory cells, cytokine levels, and ROS in BALF of mice with COPD. (a) The percentage of white blood cells in BALF. (b) The percentage of neutrophil lymphocytes in BALF. (c) The percentage of lymphocytes in BALF. (d) IL-6 level in BALF. (e) TGF-*β* level in BALF. (f) ROS level in BALF. Data were expressed as mean ± SD (*n* = 7), the experiments were repeated 3 times, and statistical significance was determined by a *t*-test. ^aa^*P* < 0.01, ^aaa^*P* < 0.001 vs control; ^b^*P* < 0.05, ^bb^*P* < 0.01, ^bbb^*P* < 0.001 vs CS + LPS group; ^c^*P* < 0.05, ^cc^*P* < 0.01 vs dexamethasone.

To evaluate the effects of luteolin on inflammatory cells, cytokine levels, and ROS in BALF, the percentage of inflammatory cells (white blood cells, neutrophils, and lymphocytes), concentrations of cytokines (IL-6, TGF-*β*), and ROS were detected. As shown in Figures 11(a)–11(c), the percentage of white blood cells, neutrophils, and lymphocytes and the concentration of IL-6 and TGF-*β* (Figures 11(d) and 11(e)) in the BALF were significantly increased in CS and LPS group compared with the control group. However, treatment with luteolin significantly decreased the percentage of white blood cells, neutrophils, and lymphocytes and the concentration of IL-6 and TGF-*β* in the BALF compared with CS and LPS group. In addition, ROS was also significantly increased in the CS and LPS group compared with the control group but decreased in the luteolin group (Figure 11(f)).

### 3.10. Effects of Luteolin on the Expressions of *α*-SMA and TGF-*β*1 in Mice with COPD

**Figure 12:**
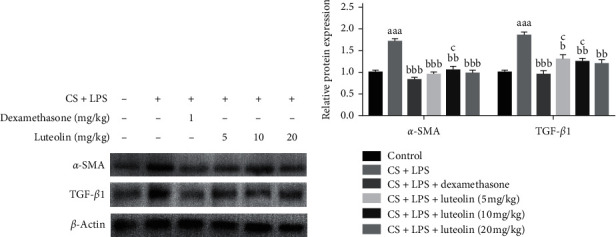
Effects of luteolin on the expressions of *α*-SMA and TGF-*β*1 in the lung. (a) Protein extracted from the lung was reacted with anti-*α*-SMA and anti-TGF-*β*1 by Western blot. (b) The relative protein expressions of *α*-SMA and TGF-*β*1. The data presents mean ± SD (*n* = 7), the experiments were repeated 3 times, and statistical significance was determined by a *t*-test. ^aaa^*P* < 0.001 vs control; ^b^*P* < 0.05, ^bb^*P* < 0.01, ^bbb^*P* < 0.001 vs CS + LPS group; ^c^*P* < 0.05 vs dexamethasone.

Resident lung fibroblasts are activated in chronic airway inflammation, accompanied by increased expression of *α*-smooth muscle actin (*α*-SMA), leading to irreversible airflow limitation. Transforming growth factor (TGF)-*β* is a well-known fibrogenic mediator involved in COPD. The expression of *α*-SMA and TGF-*β*1 in the lung was determined by western blot (Figures 12(a) and 12(b)). Elevated expression of *α*-SMA and TGF-*β*1 was observed in the CS and LPS group compared to the controls. However, the administration of luteolin markedly inhibited the expression of *α*-SMA and TGF-*β*1 in the luteolin group compared with CS and LPS group.

### 3.11. Effects of Luteolin on COPD-Related Targets

**Figure 13:**
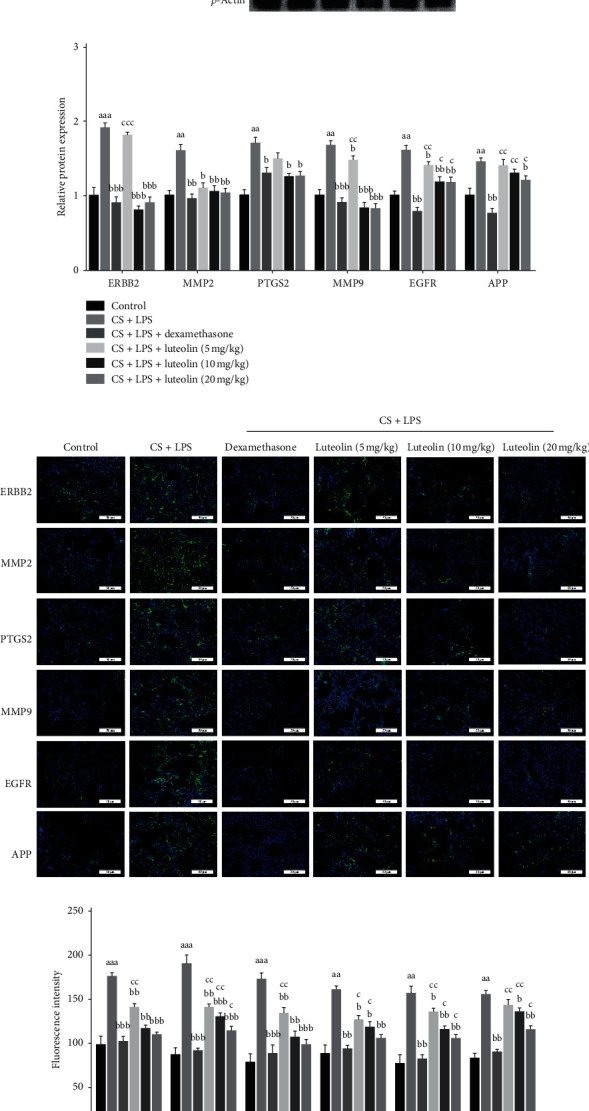
Effects of luteolin on COPD-related targets. (a) Core target-associated markers examined by western blot. (b) The relative protein expressions of core targets. (c) Core target-associated markers examined by immunofluorescence; (d) Fluorescence intensity of core targets. Data were expressed as mean ± SD (*n* = 7), the experiments were repeated 3 times, and statistical significance was determined by a *t*-test. ^aa^*P* < 0.01, ^aaa^*P* < 0.001 vs control; ^b^*P* < 0.05, ^bb^*P* < 0.01, ^bbb^*P* < 0.001 vs CS + LPS group; ^c^*P* < 0.05, ^cc^*P* < 0.01, ^ccc^*P* < 0.001 vs dexamethasone.

Western blot analyses were performed to evaluate COPD-related targets (EGFR, MMP9, PTGS2, MMP2, APP, and ERBB2) involved in the therapeutic effects of luteolin on COPD. Outcomes presented showed a significant increase in the expression of COPD-related targets in the CS and LPS group compared with the control group. However, luteolin treatment markedly inhibited the expression of COPD-related targets compared with the CS and LPS group (Figures 13(a) and 13(b)). Similarly, immunofluorescence revealed that the expression of COPD-related targets in lung tissue was significantly increased in the CS and LPS group compared with the control group, but luteolin decreased the expression of COPD-related targets compared with the CS and LPS group (Figures 13(c) and 13(d)).

## 4. Discussion

TCM has focused on the treatment of COPD for thousands of years [60]. Large-scale clinical trials have confirmed that TCM has made progress in controlling COPD [61, 62]. However, a considerable number of TCM studies exhibit defects. For example, on the one hand, the clinical efficacy evaluation is incomplete or lacks a large amount of clinical data support, the intervention time is limited, the sample size is small, and the indices of efficacy evaluation lack empirical validity. Considering the aforementioned problem, we conducted a meta-analysis. Meta-analysis aims to increase the credibility of conclusions and solve the inconsistencies of research results by increasing the sample and conducting a systematic, objective, and quantitative comprehensive analysis of multiple research results [63]. Therefore, in this article, we selected high-quality studies published in the past to evaluate the effects of TCM interventions on disease progression of COPD through meta-analysis and collected the current evidence on the total effect of SZYQD on COPD in 3730 individuals from 35 studies. The result showed that SZYQD has a curative effect on COPD.

On the other hand, in a large number of TCM have complex compounds, the active compounds are not clear in clinical and pharmacological studies, the specific targets have not fully been identified, and the mechanism of action has not been effectively elaborated. Network pharmacology illustrates the intricate interactions among genes, proteins, and metabolites related to diseases and drugs from a network perspective, which has become a powerful tool in elucidating complex and holistic mechanisms of TCM [64, 65]. Hence, we used network pharmacology to elucidate the mechanisms of multiple-target components in traditional Chinese medicine. In this study, 27 active components of SZYQD were screened through network pharmacology, among which 6 compounds, including beta-sitosterol, exceparl M-OL, luteolin, phthalic acid, butyl isohexyl ester, sinoacutine, and stigmasterol, may be the key compounds of SZYQD. Pharmacological studies have shown that *β*-sitosterol performs anti-inflammatory activity by attenuating edema in the rat model [66]. Exceparl M-OL has a certain effect on the treatment of chronic inflammatory diseases [18]. Luteolin effectively prevented LPS-induced changes in blood gas parameters and histopathology, activation of neutrophils, and infiltration of white blood cells and neutrophils into the lung [67, 68]. Phthalic acid and butyl isohexyl ester exert protective actions against lung injury and are used to treat COPD airway hyperreactivity [19]. Sinoacutine inhibits the proliferation of lung cancer cells, which may be related to the inhibition of PI3K/Akt and MAPK/ERK pathways [69]. Stigmasterol suppresses airway inflammation by inhibiting allergen-induced immunoglobulin E-mediated responses [70]. These compounds have a good correlation with the treatment of COPD. Disease-target-compound network and PPI network analysis showed that EGFR, MMP9, PTGS2, MMP2, APP, and ERBB2 may be the core targets of SZYQD in the treatment of COPD, which were mainly involved in cancer in the pathway, calcium signaling pathway, PI3K-Akt signaling pathway, and Ras signaling pathway. To further verify the key compounds of SZYQD treating COPD, we carried out molecular docking between six compounds and the core targets, and the results showed that six compounds had a good binding activity to these core targets. In particular, luteolin showed the strongest binding activity with EGFR, MMP9, PTGS2, MMP2, APP, and ERBB2.

Luteolin, an active flavonoid compound isolated from *Lonicera japonica*, is widely distributed in the plant kingdom. Preclinical studies have shown that this flavone possesses a variety of pharmacological activities, including antioxidant, anti-inflammatory, antimicrobial, and anticancer activities [71]. Recently, luteolin has been found to alleviate bronchoconstriction and airway hyperreactivity in ovalbumin sensitized mice [72]. It can also decrease inflammatory reactions in lung tissue of treated mice [72]. A study in the lipopolysaccharide (LPS) model of Ali suggested that luteolin blocked the AKT/NF-*κ*B pathway, consequently suppressing the inflammatory mediator expression [73]. Liu et al. [74] studied that luteolin may be an effective candidate for the treatment of HgCl2-induced lung injury by preventing NF-*κ*B activation and activating AKT/Nrf2 pathway. A study also showed that luteolin attenuates neutrophilic oxidative stress and inflammatory arthritis by inhibiting Raf1 activity [68].

Although luteolin has a broad application prospect in the treatment of lung diseases, inflammation, and oxidative stress process, there are few reports about its treatment of COPD. Thus, we used CS and LPS to establish a COPD mice model, exploring the effects of luteolin on COPD induced by CS and LPS. Results presented a reduction of tidal volume and lung injury under CS and LPS stimulation; meanwhile, treatment with luteolin prominently improved this condition. Additionally, we found that the increase in the number of inflammatory cells in the BAL fluid after CS and LPS stimulation was significantly attenuated by luteolin. Luteolin also reduced the production of IL-6, TGF-*β*, ROS, and histological signs of pulmonary fibrosis including such as *α*-SMA and TGF-*β*1. Our study also revealed that the protein expressions of COPD-related targets such as EGFR, MMP9, PTGS2, MMP2, APP, and ERBB2 in lung tissue were significantly increased in the COPD group and were prevented in the luteolin group. Although network pharmacology provides a rough direction, substantial experimental data are necessary for verification. Luteolin is a candidate compound for COPD treatment by regulating EGFR, MMP9, PTGS2, MMP2, APP, and ERBB2 through a preliminary decomposition experiment.

## 5. Conclusion

SZYQD has a curative effect on COPD, and its mechanism is related to 27 compounds, 104 targets, and 35 pathways. Meanwhile, luteolin is a candidate compound for COPD treatment by regulating EGFR, MMP9, PTGS2, MMP2, APP, and ERBB2. The strategy of integrating classical pharmacology with systems pharmacology analysis has the potential to provide a better strategy for the understanding of the TCM mechanism.

## Figures and Tables

**Table 1:** The characteristics of all included studies.

Author and reference	Year	Total sample	Control/treatment	Period of treatment (days)	Drug		Evaluation standard
					*C*	*T*	
Cui et al. [26]	2017	100	50/50	7 d	1	2	①
Deng and Ceng [27]	2017	102	51/51	—	1	2	①
Deng and Tan [28]	2020	80	40/40	84 d	1	2	①②③
Gong and Shen [29]	2014	88	44/44	14 d	1	2	①④
He et al. [30]	2018	94	47/47	10 d	1	2	①②③
Huang [31]	2019	94	47/47	14 d	1	2	①②③
Hui [32]	2019	90	45/45	7 d	1	2	①③
Lai et al. [33]	2017	80	40/40	14 d	1	2	①
Li [34]	2014	88	44/44	14 d	1	2	①
Li et al. [35]	2019	180	90/90	7 d	1	2	①③
Li [36]	2020	97	48/49	12 d	1	2	①
Li et al. [37]	2013	112	56/56	28 d	1	2	①③
Li et al. [38]	2018	150	75/75	15 d	1	2	①
Liu [39]	2015	84	42/42	—	1	2	①
Liu [40]	2019	80	40/40	12 d	1	2	①
Lu and Wang [41]	2018	160	80/80	30 d	1	2	①④⑤
Lu [42]	2019	80	40/40	14 d	1	2	①②③
Lv et al. [43]	2011	100	50/50	14 d	1	2	①
Pang [44]	2019	82	41/41	7 d	1	2	①
Tian et al. [45]	2005	99	49/50	30 d	1	2	①
Wang [46]	2019	96	48/48	90 d	1	2	①②③
Wang et al. [47]	2020	84	42/42	14d	1	2	①②③⑤
Wu [48]	2018	252	126/126	—	1	2	①
Xu [49]	2020	80	40/40	14 d	1	2	①
Yan [50]	2018	80	40/40	15 d	1	2	①④⑤
Yang [51]	2011	160	72/88	14 d	1	2	①
Yang and Shi [52]	2014	86	40/46	14 d	1	2	①②③④⑤
Yang [53]	2020	80	40/40	90 d	1	2	①③
Yuan et al. [54]	2014	127	63/64	14 d	1	2	①②③
Zhang [55]	2016	100	50/50	15 d	1	2	①②③
Zhang [56]	2011	80	40/40	30 d	1	2	①
Zhang et al. [57]	2013	120	62/58	7 d	1	2	①④⑤
Zhang [14]	2018	80	38/42	7 d	1	2	①
Zhang and Fang [58]	2006	85	37/48	28 d	1	2	①②③
Zhou and Huang [59]	2017	160	80/80	30 d	1	2	②

*Note.* 1: routine western medicine treatment (including oxygen absorption, anti-infection, phlegm reduction, bronchial dilator, and glucocorticoid.); 2: Sanzi Yangqin decoction based on other medicine or Sanzi Yangqin decoction alone. ① Clinical efficacy. ② FEV1%. ③ FEV1%/FVC. ④ PaO2. ⑤ PaCO2.

**Table 2:** Molecular docking of main active compounds of SZYQD and core targets.

Target name	PDB ID	Compound	Energy (kcal/mol)
EGFR	5y9t	Beta-sitosterol	−5.28
EGFR	5y9t	Exceparl M-OL	−5.24
EGFR	5y9t	Luteolin	−7.16
EGFR	5y9t	Phthalic acid, butyl isohexyl ester	−5.19
EGFR	5y9t	Sinoacutine	−5.26
EGFR	5y9t	Stigmasterol	−6.27
MMP9	5th9	Beta-sitosterol	−5.28
MMP9	5th9	Exceparl M-OL	−6.00
MMP9	5th9	Luteolin	−8.4
MMP9	5th9	Phthalic acid, butyl isohexyl ester	−5.84
MMP9	5th9	Sinoacutine	−5.82
MMP9	5th9	Stigmasterol	−6.02
MMP2	3ayu	Beta-sitosterol	−5.12
MMP2	3ayu	Exceparl M-OL	−5.48
MMP2	3ayu	Luteolin	−7.21
MMP2	3ayu	Phthalic acid, butyl isohexyl ester	−6.24
MMP2	3ayu	Sinoacutine	−5.82
MMP2	3ayu	Stigmasterol	−6.25
APP	3ktm	Beta-sitosterol	−5.23
APP	3ktm	Exceparl M-OL	−5.71
APP	3ktm	Luteolin	−7.00
APP	3ktm	Phthalic acid, butyl isohexyl ester	−5.30
APP	3ktm	Sinoacutine	−6.77
APP	3ktm	Stigmasterol	−5.34
ERBB2	3wlw	Beta-sitosterol	−5.87
ERBB2	3wlw	Exceparl M-OL	−5.53
ERBB2	3wlw	Luteolin	−7.10
ERBB2	3wlw	Phthalic acid, butyl isohexyl ester	−5.33
ERBB2	3wlw	Sinoacutine	−5.02
ERBB2	3wlw	Stigmasterol	−5.68
PTGS2	5kir	Beta-sitosterol	−6.12
PTGS2	5kir	Exceparl M-OL	−5.89
PTGS2	5kir	Luteolin	−7.50
PTGS2	5kir	Phthalic acid, butyl isohexyl ester	−5.35
PTGS2	5kir	Sinoacutine	−5.21
PTGS2	5kir	Stigmasterol	−6.82

*Note.* Docking scores of main active compounds of SZYQD in the disease-compound-target network and the top 6-degree value of the target genes in the PPI network. The lower the energy of binding with receptors is, the greater the possibility of action is.

## Data Availability

All data used to support the findings of this study are included within the article.

## Supplementary Materials

Supplementary Materials Supplementary File 1: information of 27 active compounds of Sanzi Yangqin decoction and degree value of corresponding compounds. Supplementary File 2: a component-compound-target network of white mustard seed (A), radish seed (B), and perilla seed (C). The red nodes represent the targets, the green nodes represent the compounds, while the yellow nodes represent the components and those compounds are linked with the corresponding targets.
