# Supplementary material for: The Clinical Efficiency and the Mechanism of Sanzi Yangqin Decoction for Chronic Obstructive Pulmonary Disease
Source: Evid Based Complement Alternat Med. 2021 Jun 10;2021:5565562. doi: 10.1155/2021/5565562 (PMC8213503; doi:10.1155/2021/5565562)
Supplement: Supplementary Materials — Supplementary File 1: information of 27 active compounds of Sanzi Yangqin decoction and degree value of corresponding compounds. Supplementary File 2: a component-compound-target network of white mustard seed (A), radish seed (B), and perilla seed (C). The red nodes represent the targets, the green nodes represent the compounds, while the yellow nodes represent the components and those compounds are linked with the corresponding targets. [file 5565562.f1.zip › 5565562.f1/Supplement File 2.docx]

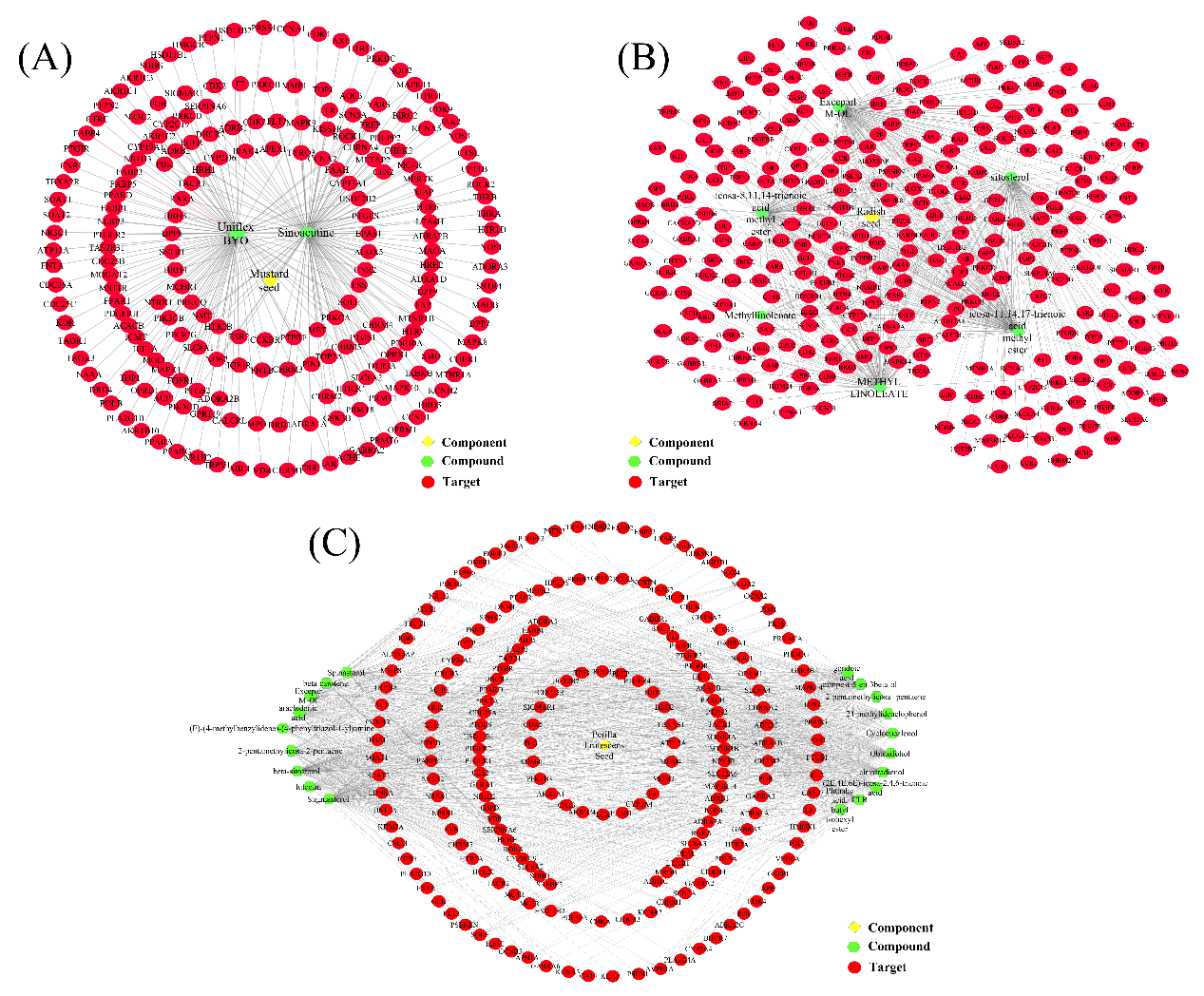


**Component-compound-target network of white mustard seed (A), radish seed (B) and perilla seed (C).** The red nodes represent the targets, the green nodes represent the compounds while the yellow nodes represent the components and those compounds are linked with the corresponding targets.
